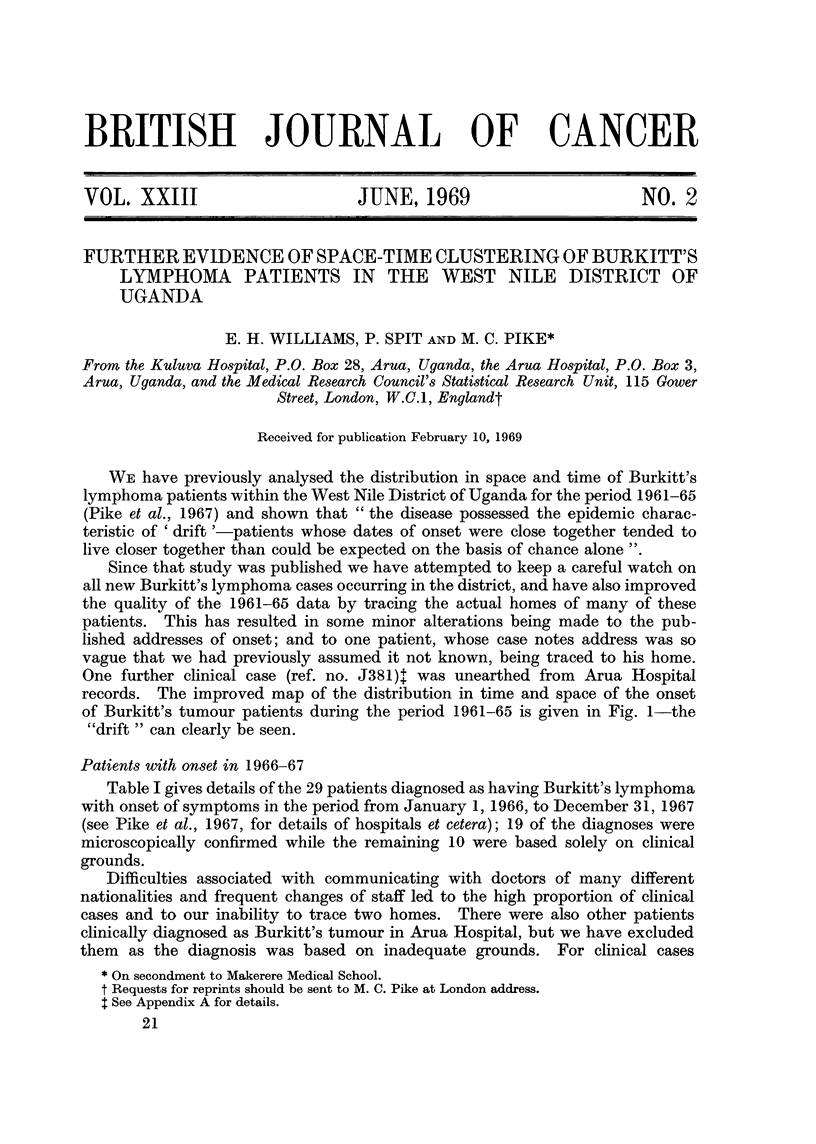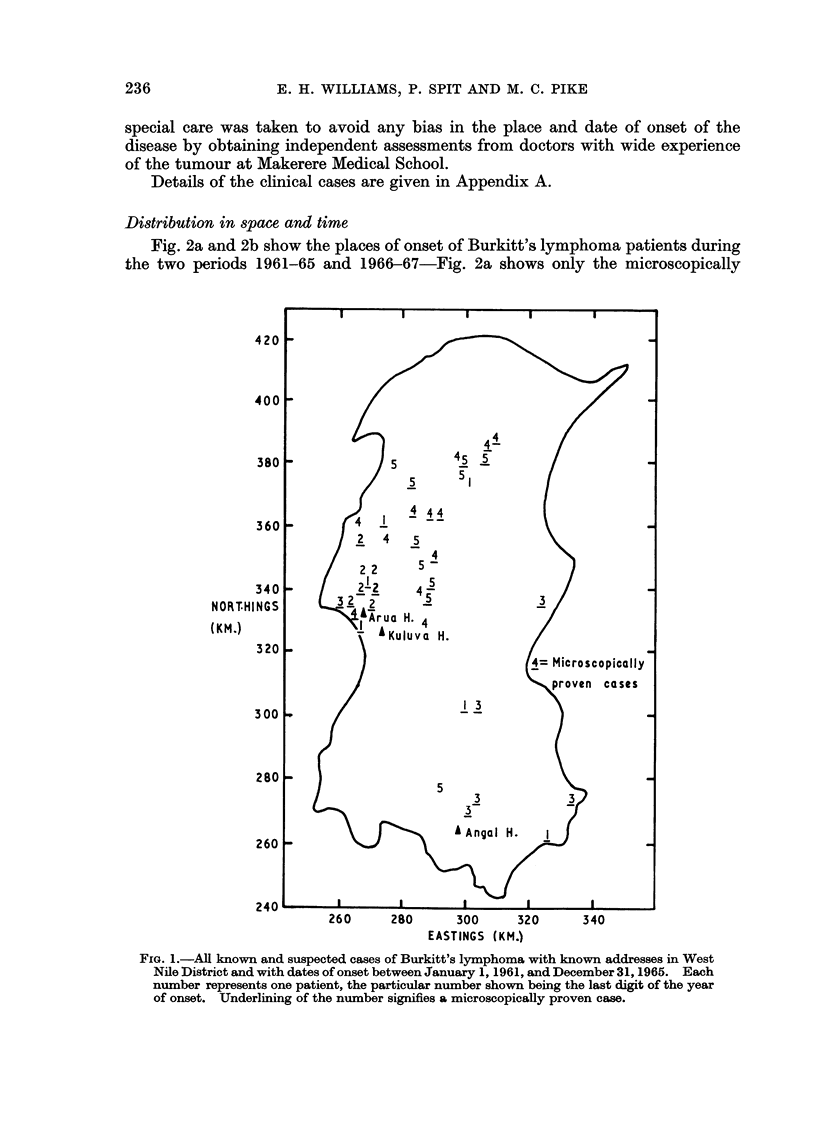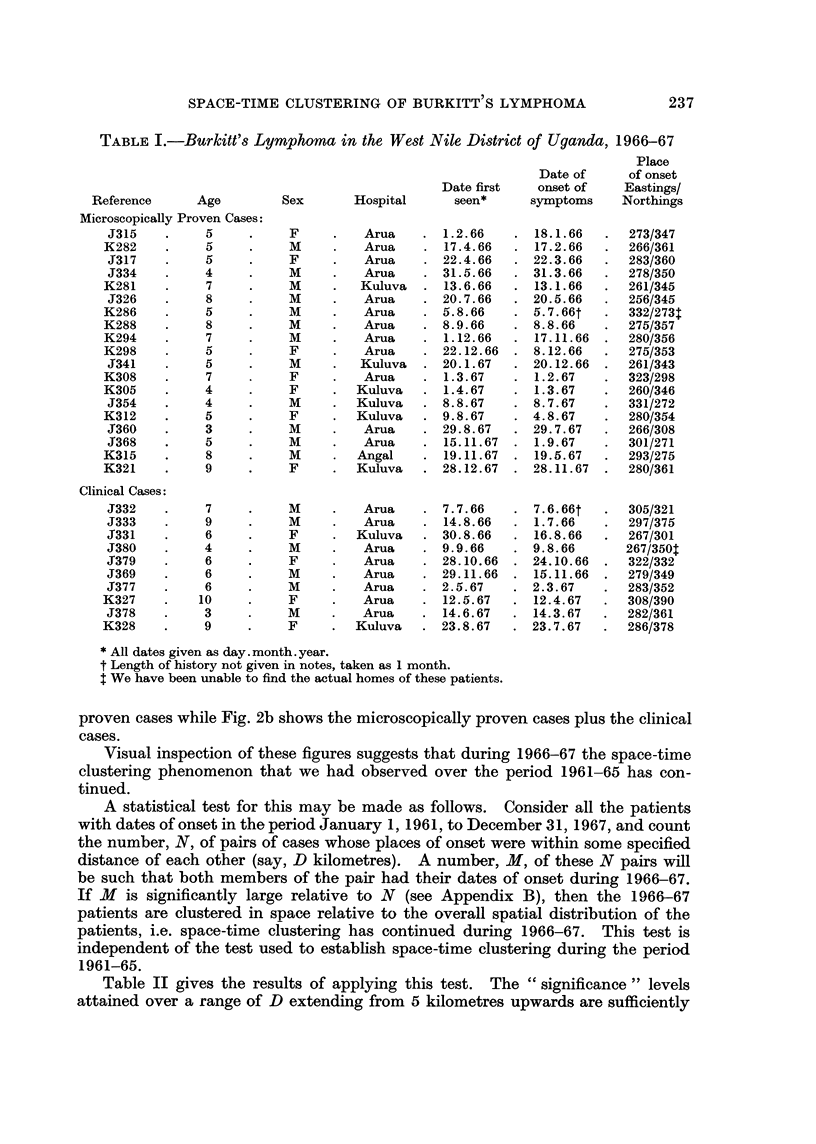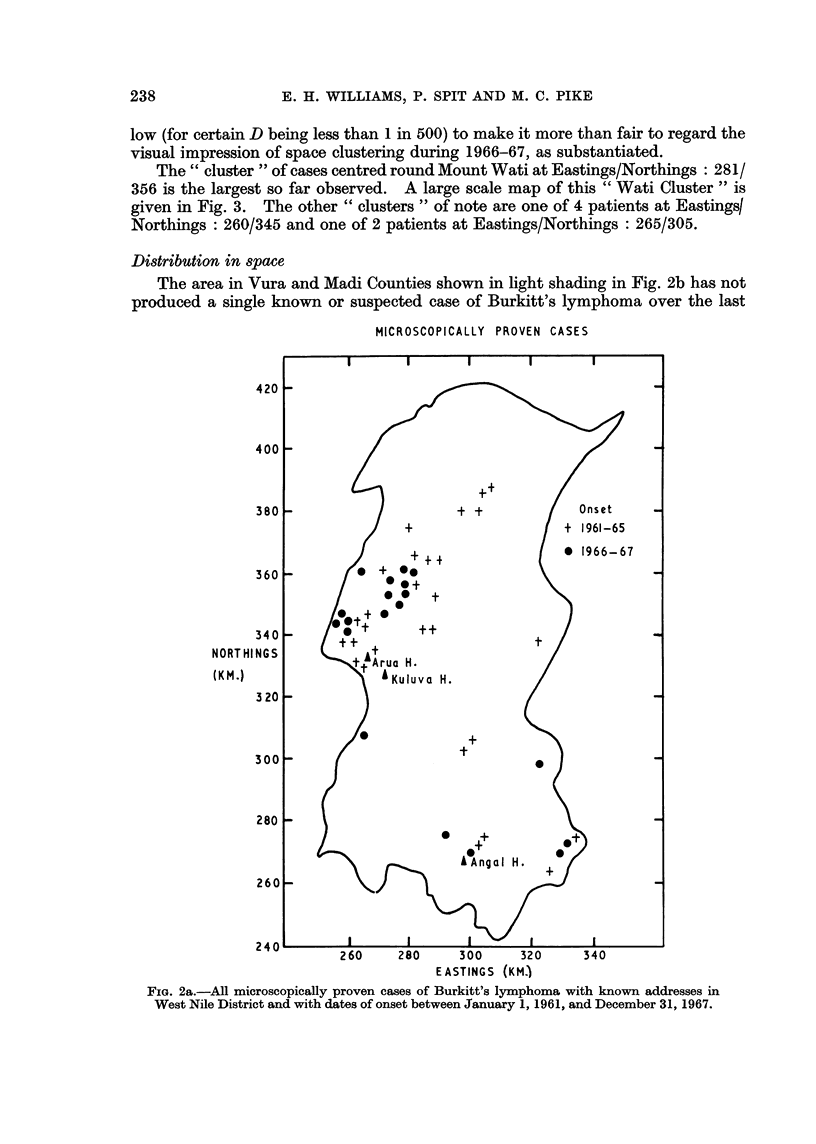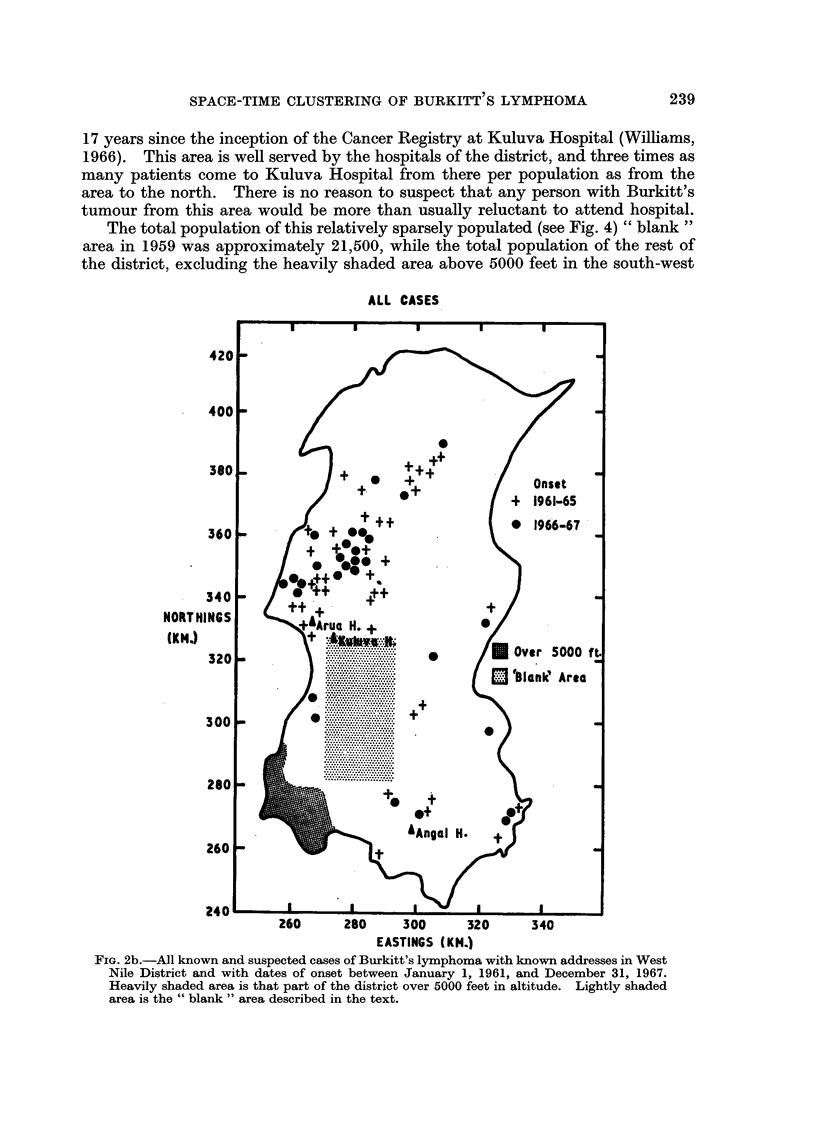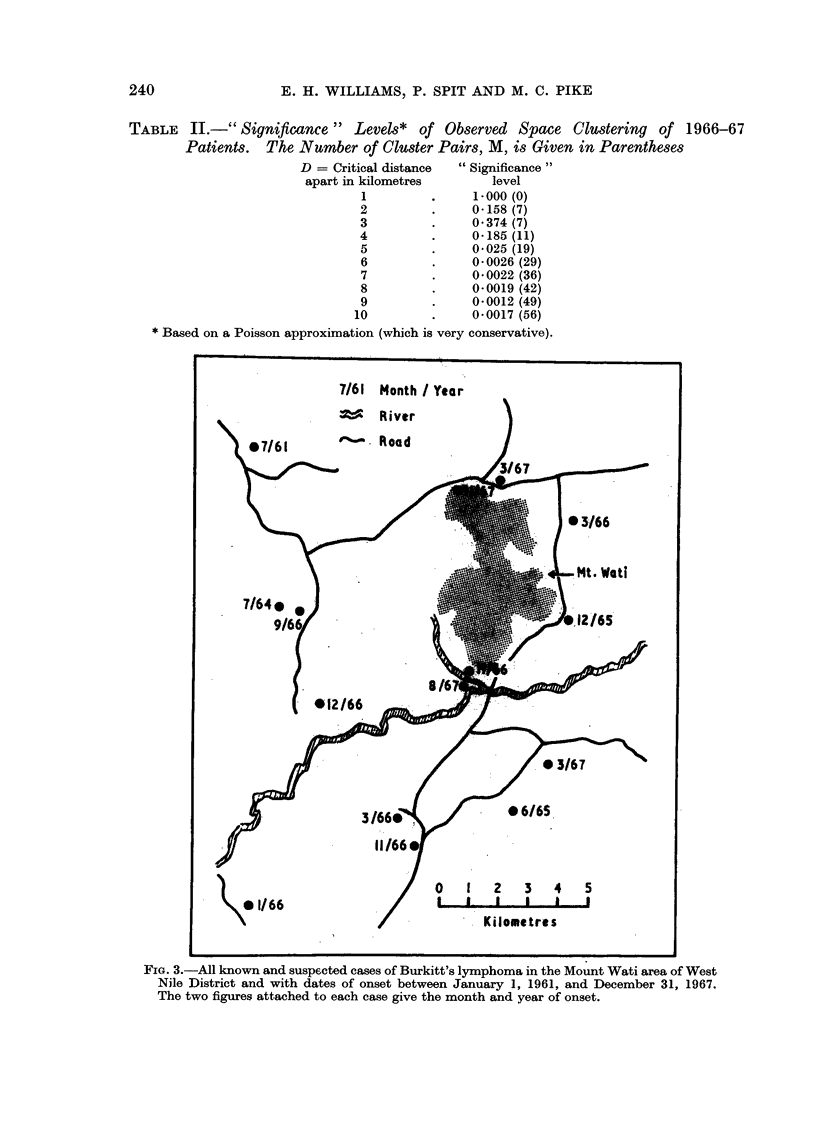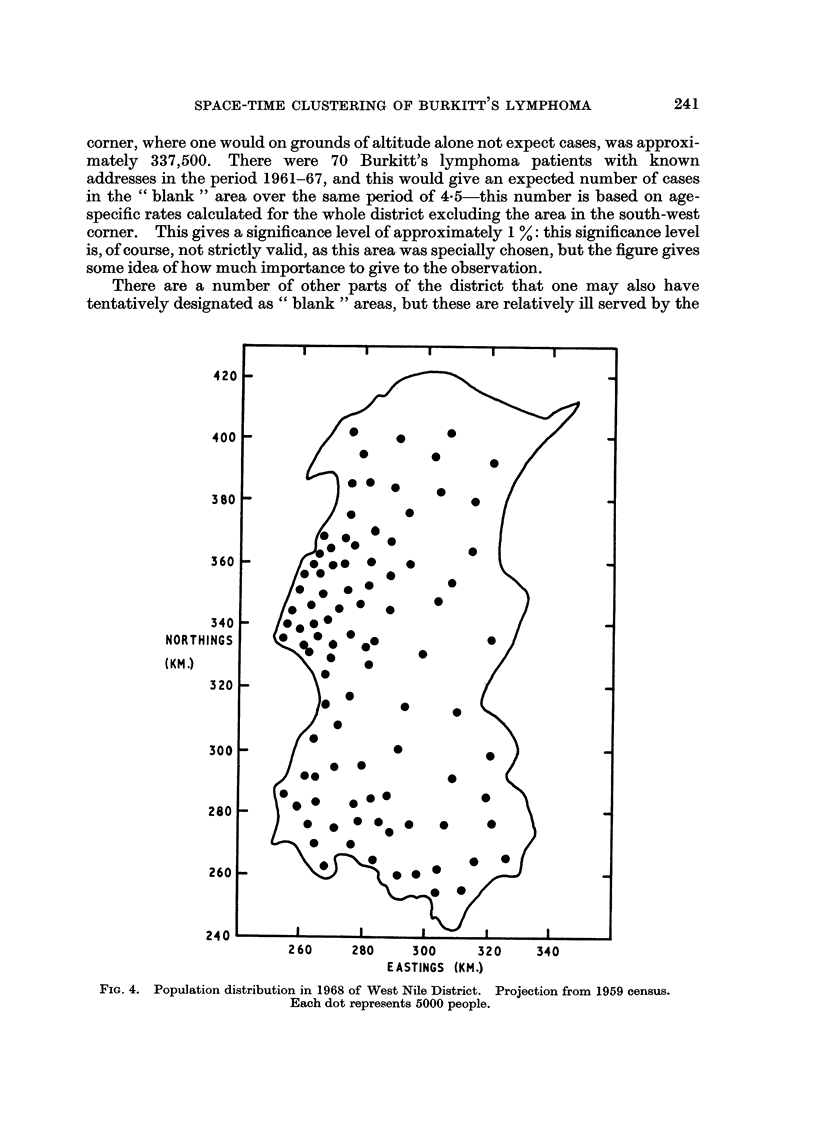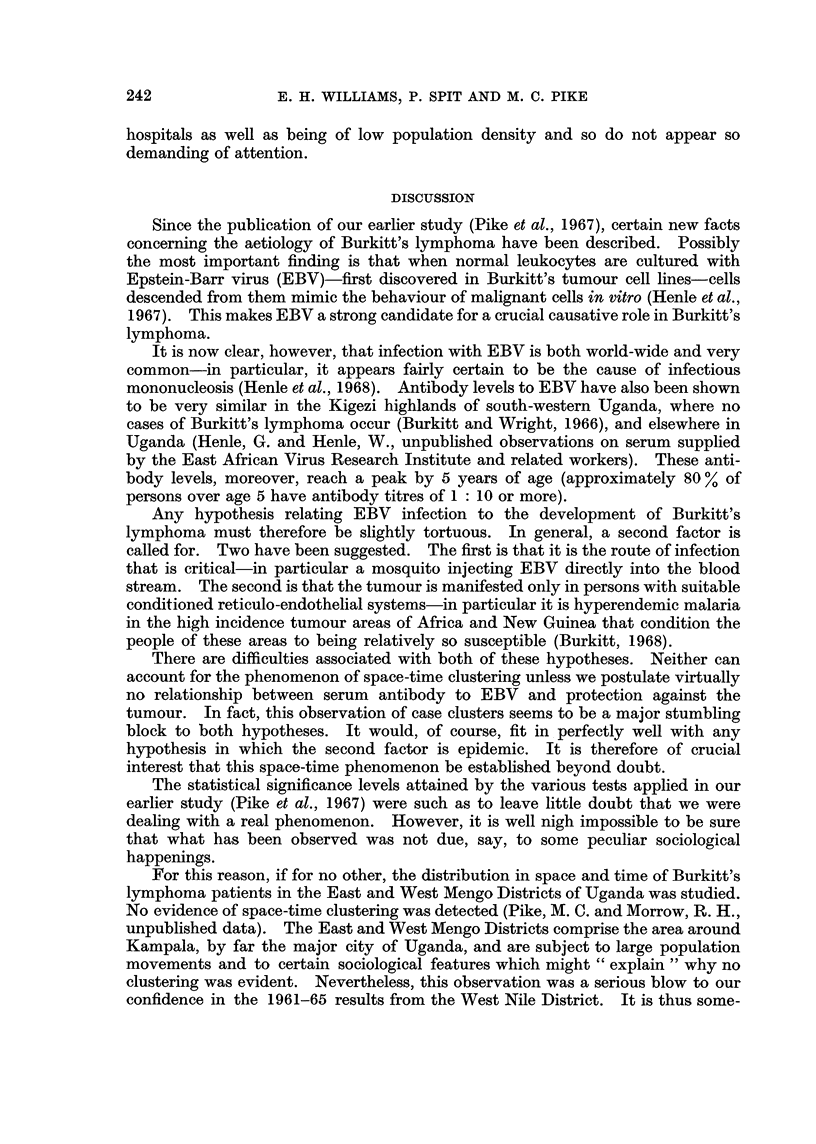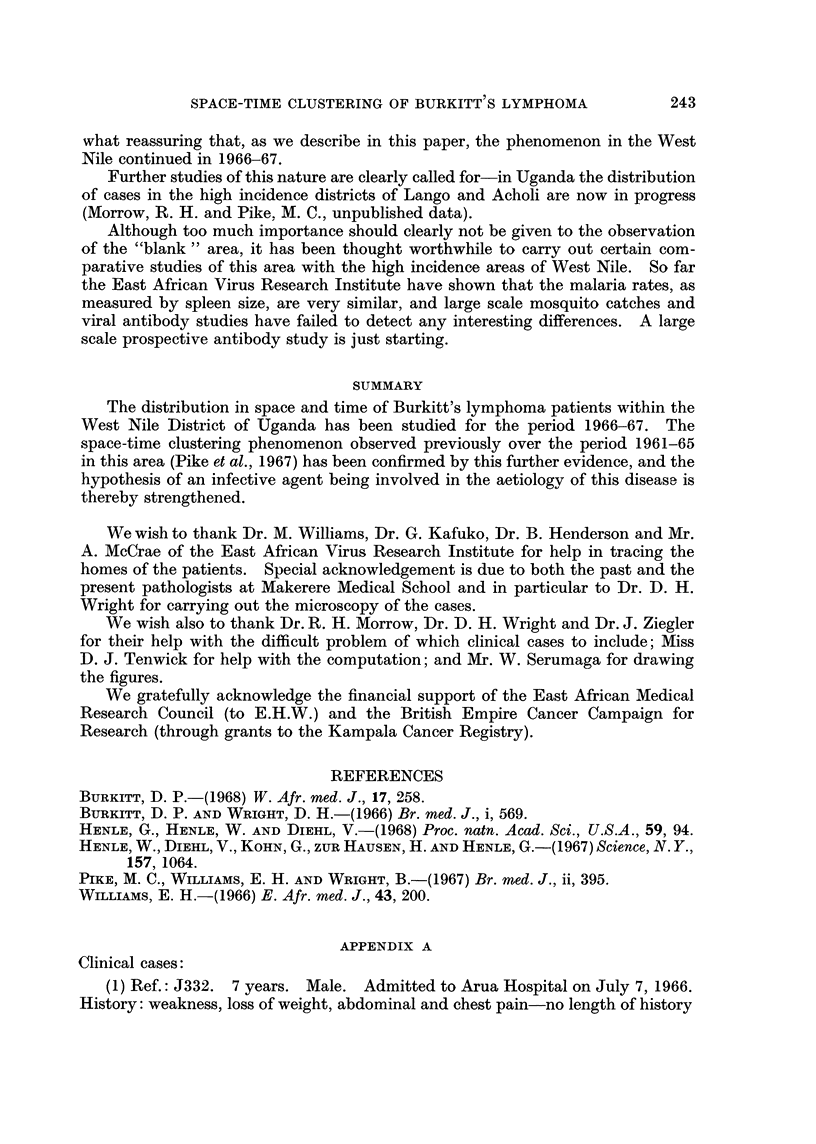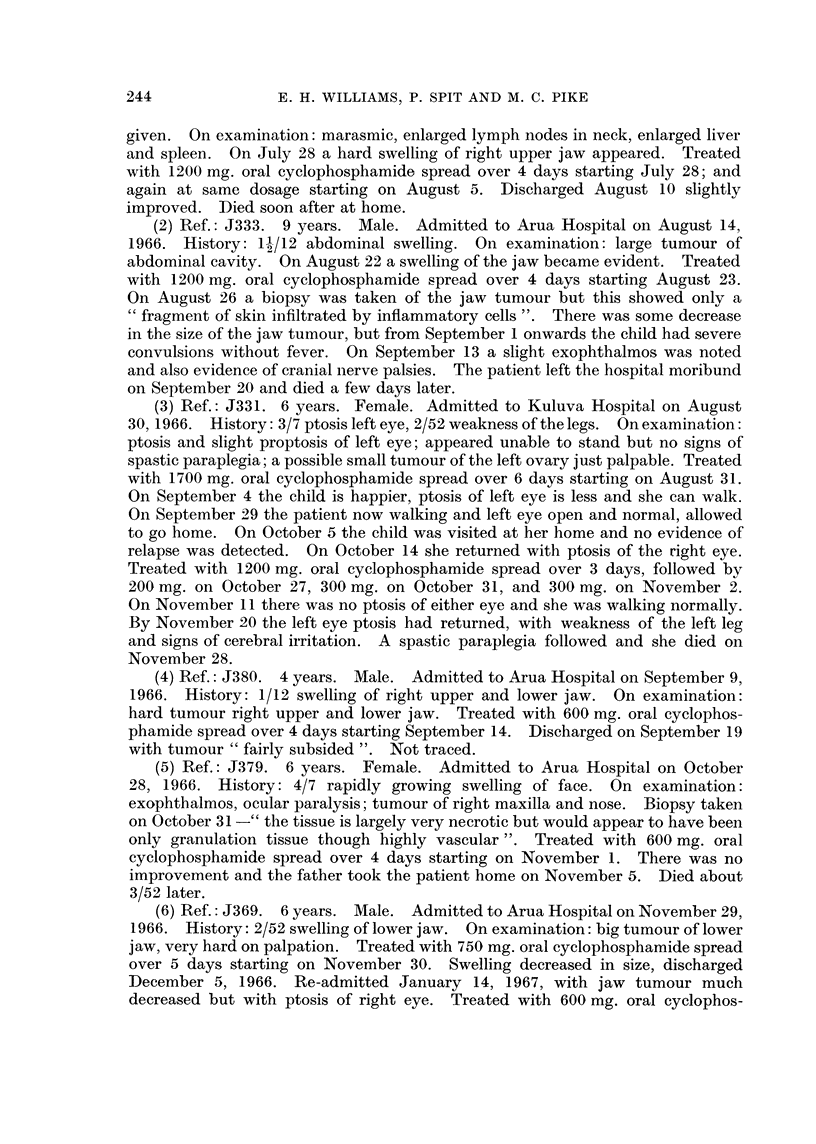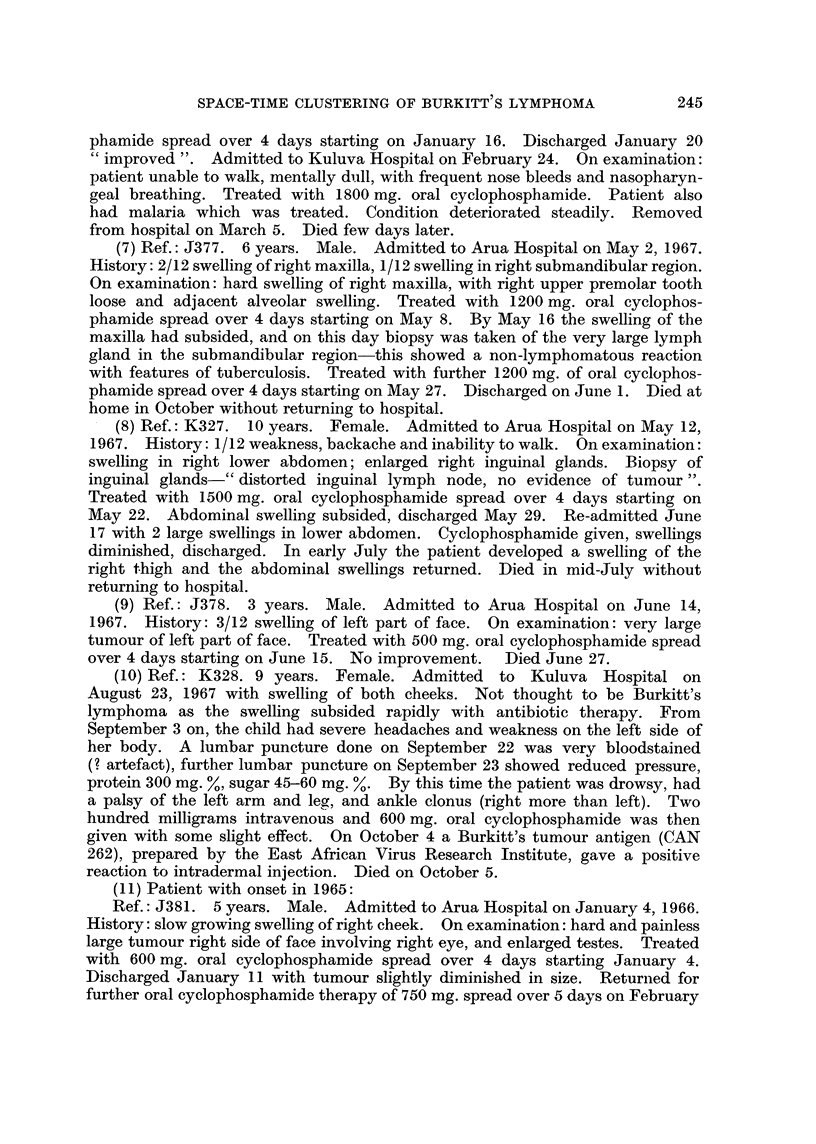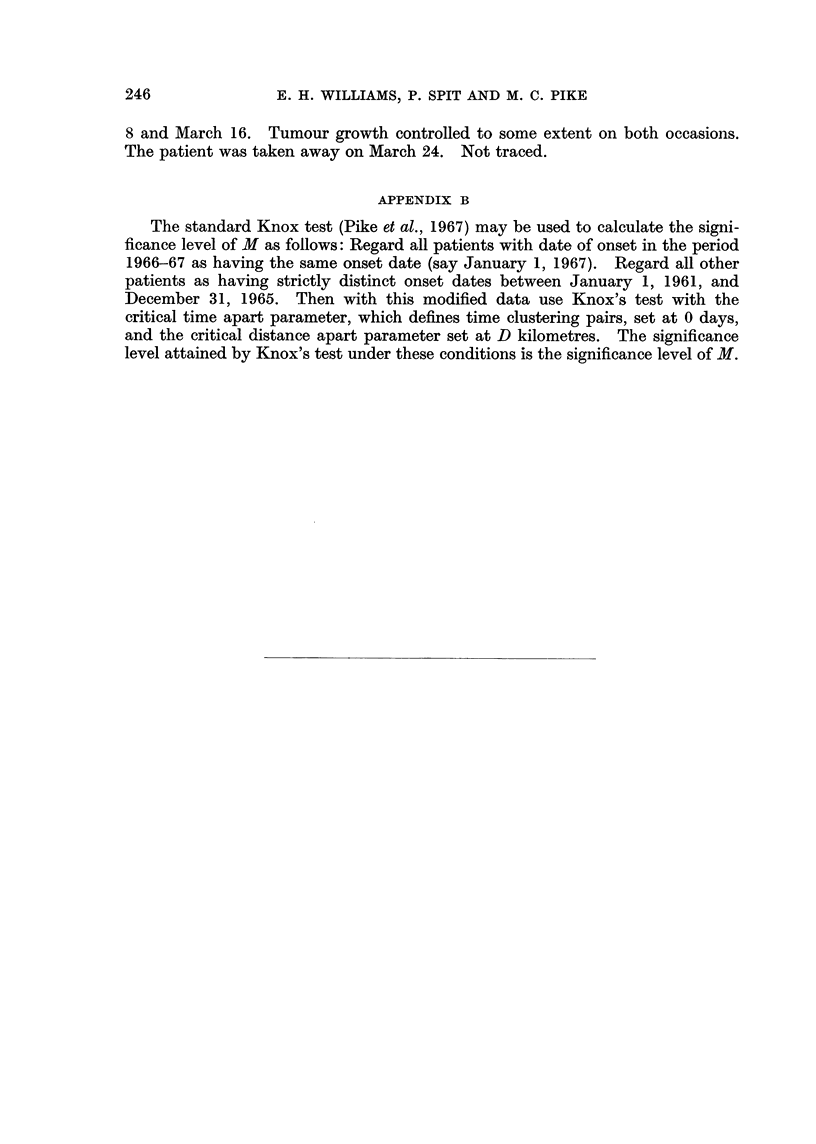# Further evidence of space-time clustering of Burkitt's lymphoma patients in the West Nile District of Uganda.

**DOI:** 10.1038/bjc.1969.33

**Published:** 1969-06

**Authors:** E. H. Williams, P. Spit, M. C. Pike


### BRITISH JOURNAL OF CANCER

VOL. XXIII JUNE, 1969 NO. 2

### FURTHER EVIDENCE OF SPACE-TIME CLUSTERING OF BURKITT'S LYMPHOMA PATIENTS IN THE WEST NILE DISTRICT OF UGANDA

E. H. WILLIAMS, P. SPIT AND M. C. PIKE*

*From the Kuluva Hospital, P.O. Box* 28, *Arua, Uganda, the Arua Hospital, P.O. Box* 3, *Arua, Uganda, and the Medical Research Council's Statistical Research Unit,* 115 *Gower Street, London, W.C.*1, *England*†

Received for publication February 10, 1969

WE have previously analysed the distribution in space and time of Burkitt's lymphoma patients within the West Nile District of Uganda for the period 1961–65 (Pike *et al.*, 1967) and shown that " the disease possessed the epidemic characteristic of ' drift '—patients whose dates of onset were close together tended to live closer together than could be expected on the basis of chance alone ".

Since that study was published we have attempted to keep a careful watch on all new Burkitt's lymphoma cases occurring in the district, and have also improved the quality of the 1961–65 data by tracing the actual homes of many of these patients. This has resulted in some minor alterations being made to the published addresses of onset; and to one patient, whose case notes address was so vague that we had previously assumed it not known, being traced to his home. One further clinical case (ref. no. J381)‡ was unearthed from Arua Hospital records. The improved map of the distribution in time and space of the onset of Burkitt's tumour patients during the period 1961–65 is given in Fig. 1—the "drift " can clearly be seen.

*Patients with onset in* 1966–67

Table I gives details of the 29 patients diagnosed as having Burkitt's lymphoma with onset of symptoms in the period from January 1, 1966, to December 31, 1967 (see Pike *et al.*, 1967, for details of hospitals *et cetera*); 19 of the diagnoses were microscopically confirmed while the remaining 10 were based solely on clinical grounds.

Difficulties associated with communicating with doctors of many different nationalities and frequent changes of staff led to the high proportion of clinical cases and to our inability to trace two homes. There were also other patients clinically diagnosed as Burkitt's tumour in Arua Hospital, but we have excluded them as the diagnosis was based on inadequate grounds. For clinical cases

* On secondment to Makerere Medical School.
† Requests for reprints should be sent to M. C. Pike at London address.
‡ See Appendix A for details.

special care was taken to avoid any bias in the place and date of onset of the disease by obtaining independent assessments from doctors with wide experience of the tumour at Makerere Medical School.

Details of the clinical cases are given in Appendix A.

*Distribution in space and time*

Fig. 2a and 2b show the places of onset of Burkitt's lymphoma patients during the two periods 1961–65 and 1966–67—Fig. 2a shows only the microscopically

Fig. 1.—All known and suspected cases of Burkitt's lymphoma with known addresses in West Nile District and with dates of onset between January 1, 1961, and December 31, 1965. Each number represents one patient, the particular number shown being the last digit of the year of onset. Underlining of the number signifies a microscopically proven case.

TABLE I.—*Burkitt's Lymphoma in the West Nile District of Uganda*, 1966–67

| Reference | Age | Sex | Hospital | Date first seen* | Date of onset of symptoms | Place of onset Eastings/ Northings |
|---|---|---|---|---|---|---|
| Microscopically Proven Cases: | | | | | | |
| J315 | 5 | F | Arua | 1.2.66 | 18.1.66 | 273/347 |
| K282 | 5 | M | Arua | 17.4.66 | 17.2.66 | 266/361 |
| J317 | 5 | F | Arua | 22.4.66 | 22.3.66 | 283/360 |
| J334 | 4 | M | Arua | 31.5.66 | 31.3.66 | 278/350 |
| K281 | 7 | M | Kuluva | 13.6.66 | 13.1.66 | 261/345 |
| J326 | 8 | M | Arua | 20.7.66 | 20.5.66 | 256/345 |
| K286 | 5 | M | Arua | 5.8.66 | 5.7.66† | 332/273‡ |
| K288 | 8 | M | Arua | 8.9.66 | 8.8.66 | 275/357 |
| K294 | 7 | M | Arua | 1.12.66 | 17.11.66 | 280/356 |
| K298 | 5 | F | Arua | 22.12.66 | 8.12.66 | 275/353 |
| J341 | 5 | M | Kuluva | 20.1.67 | 20.12.66 | 261/343 |
| K308 | 7 | F | Arua | 1.3.67 | 1.2.67 | 323/298 |
| K305 | 4 | F | Kuluva | 1.4.67 | 1.3.67 | 260/346 |
| J354 | 4 | M | Kuluva | 8.8.67 | 8.7.67 | 331/272 |
| K312 | 5 | F | Kuluva | 9.8.67 | 4.8.67 | 280/354 |
| J360 | 3 | M | Arua | 29.8.67 | 29.7.67 | 266/308 |
| J368 | 5 | M | Arua | 15.11.67 | 1.9.67 | 301/271 |
| K315 | 8 | M | Angal | 19.11.67 | 19.5.67 | 293/275 |
| K321 | 9 | F | Kuluva | 28.12.67 | 28.11.67 | 280/361 |
| Clinical Cases: | | | | | | |
| J332 | 7 | M | Arua | 7.7.66 | 7.6.66† | 305/321 |
| J333 | 9 | M | Arua | 14.8.66 | 1.7.66 | 297/375 |
| J331 | 6 | F | Kuluva | 30.8.66 | 16.8.66 | 267/301 |
| J380 | 4 | M | Arua | 9.9.66 | 9.8.66 | 267/350‡ |
| J379 | 6 | F | Arua | 28.10.66 | 24.10.66 | 322/332 |
| J369 | 6 | M | Arua | 29.11.66 | 15.11.66 | 279/349 |
| J377 | 6 | M | Arua | 2.5.67 | 2.3.67 | 283/352 |
| K327 | 10 | F | Arua | 12.5.67 | 12.4.67 | 308/390 |
| J378 | 3 | M | Arua | 14.6.67 | 14.3.67 | 282/361 |
| K328 | 9 | F | Kuluva | 23.8.67 | 23.7.67 | 286/378 |

* All dates given as day.month.year.
† Length of history not given in notes, taken as 1 month.
‡ We have been unable to find the actual homes of these patients.

proven cases while Fig. 2b shows the microscopically proven cases plus the clinical cases.

Visual inspection of these figures suggests that during 1966–67 the space-time clustering phenomenon that we had observed over the period 1961–65 has continued.

A statistical test for this may be made as follows. Consider all the patients with dates of onset in the period January 1, 1961, to December 31, 1967, and count the number, $N$, of pairs of cases whose places of onset were within some specified distance of each other (say, $D$ kilometres). A number, $M$, of these $N$ pairs will be such that both members of the pair had their dates of onset during 1966–67. If $M$ is significantly large relative to $N$ (see Appendix B), then the 1966–67 patients are clustered in space relative to the overall spatial distribution of the patients, i.e. space-time clustering has continued during 1966–67. This test is independent of the test used to establish space-time clustering during the period 1961–65.

Table II gives the results of applying this test. The " significance " levels attained over a range of $D$ extending from 5 kilometres upwards are sufficiently low (for certain $D$ being less than 1 in 500) to make it more than fair to regard the visual impression of space clustering during 1966–67, as substantiated.

The " cluster " of cases centred round Mount Wati at Eastings/Northings : 281/356 is the largest so far observed. A large scale map of this " Wati Cluster " is given in Fig. 3. The other " clusters " of note are one of 4 patients at Eastings/Northings : 260/345 and one of 2 patients at Eastings/Northings : 265/305.

*Distribution in space*

The area in Vura and Madi Counties shown in light shading in Fig. 2b has not produced a single known or suspected case of Burkitt's lymphoma over the last

FIG. 2a.—All microscopically proven cases of Burkitt's lymphoma with known addresses in West Nile District and with dates of onset between January 1, 1961, and December 31, 1967.

17 years since the inception of the Cancer Registry at Kuluva Hospital (Williams, 1966). This area is well served by the hospitals of the district, and three times as many patients come to Kuluva Hospital from there per population as from the area to the north. There is no reason to suspect that any person with Burkitt's tumour from this area would be more than usually reluctant to attend hospital.

The total population of this relatively sparsely populated (see Fig. 4) " blank " area in 1959 was approximately 21,500, while the total population of the rest of the district, excluding the heavily shaded area above 5000 feet in the south-west

FIG. 2b.—All known and suspected cases of Burkitt's lymphoma with known addresses in West Nile District and with dates of onset between January 1, 1961, and December 31, 1967. Heavily shaded area is that part of the district over 5000 feet in altitude. Lightly shaded area is the " blank " area described in the text.

TABLE II.—"*Significance*" *Levels* of Observed Space Clustering of* 1966–67 *Patients. The Number of Cluster Pairs*, M, *is Given in Parentheses*

| $D$ = Critical distance apart in kilometres | | "Significance" level |
|---|---|---|
| 1 | . | 1·000 (0) |
| 2 | . | 0·158 (7) |
| 3 | . | 0·374 (7) |
| 4 | . | 0·185 (11) |
| 5 | . | 0·025 (19) |
| 6 | . | 0·0026 (29) |
| 7 | . | 0·0022 (36) |
| 8 | . | 0·0019 (42) |
| 9 | . | 0·0012 (49) |
| 10 | . | 0·0017 (56) |

* Based on a Poisson approximation (which is very conservative).

FIG. 3.—All known and suspected cases of Burkitt's lymphoma in the Mount Wati area of West Nile District and with dates of onset between January 1, 1961, and December 31, 1967. The two figures attached to each case give the month and year of onset.

corner, where one would on grounds of altitude alone not expect cases, was approximately 337,500. There were 70 Burkitt's lymphoma patients with known addresses in the period 1961–67, and this would give an expected number of cases in the " blank " area over the same period of 4·5—this number is based on age-specific rates calculated for the whole district excluding the area in the south-west corner. This gives a significance level of approximately 1 %: this significance level is, of course, not strictly valid, as this area was specially chosen, but the figure gives some idea of how much importance to give to the observation.

There are a number of other parts of the district that one may also have tentatively designated as " blank " areas, but these are relatively ill served by the

Fig. 4. Population distribution in 1968 of West Nile District. Projection from 1959 census. Each dot represents 5000 people.

hospitals as well as being of low population density and so do not appear so demanding of attention.

### DISCUSSION

Since the publication of our earlier study (Pike *et al.*, 1967), certain new facts concerning the aetiology of Burkitt's lymphoma have been described. Possibly the most important finding is that when normal leukocytes are cultured with Epstein-Barr virus (EBV)—first discovered in Burkitt's tumour cell lines—cells descended from them mimic the behaviour of malignant cells *in vitro* (Henle *et al.*, 1967). This makes EBV a strong candidate for a crucial causative role in Burkitt's lymphoma.

It is now clear, however, that infection with EBV is both world-wide and very common—in particular, it appears fairly certain to be the cause of infectious mononucleosis (Henle *et al.*, 1968). Antibody levels to EBV have also been shown to be very similar in the Kigezi highlands of south-western Uganda, where no cases of Burkitt's lymphoma occur (Burkitt and Wright, 1966), and elsewhere in Uganda (Henle, G. and Henle, W., unpublished observations on serum supplied by the East African Virus Research Institute and related workers). These antibody levels, moreover, reach a peak by 5 years of age (approximately 80% of persons over age 5 have antibody titres of 1 : 10 or more).

Any hypothesis relating EBV infection to the development of Burkitt's lymphoma must therefore be slightly tortuous. In general, a second factor is called for. Two have been suggested. The first is that it is the route of infection that is critical—in particular a mosquito injecting EBV directly into the blood stream. The second is that the tumour is manifested only in persons with suitable conditioned reticulo-endothelial systems—in particular it is hyperendemic malaria in the high incidence tumour areas of Africa and New Guinea that condition the people of these areas to being relatively so susceptible (Burkitt, 1968).

There are difficulties associated with both of these hypotheses. Neither can account for the phenomenon of space-time clustering unless we postulate virtually no relationship between serum antibody to EBV and protection against the tumour. In fact, this observation of case clusters seems to be a major stumbling block to both hypotheses. It would, of course, fit in perfectly well with any hypothesis in which the second factor is epidemic. It is therefore of crucial interest that this space-time phenomenon be established beyond doubt.

The statistical significance levels attained by the various tests applied in our earlier study (Pike *et al.*, 1967) were such as to leave little doubt that we were dealing with a real phenomenon. However, it is well nigh impossible to be sure that what has been observed was not due, say, to some peculiar sociological happenings.

For this reason, if for no other, the distribution in space and time of Burkitt's lymphoma patients in the East and West Mengo Districts of Uganda was studied. No evidence of space-time clustering was detected (Pike, M. C. and Morrow, R. H., unpublished data). The East and West Mengo Districts comprise the area around Kampala, by far the major city of Uganda, and are subject to large population movements and to certain sociological features which might " explain " why no clustering was evident. Nevertheless, this observation was a serious blow to our confidence in the 1961–65 results from the West Nile District. It is thus somewhat reassuring that, as we describe in this paper, the phenomenon in the West Nile continued in 1966–67.

Further studies of this nature are clearly called for—in Uganda the distribution of cases in the high incidence districts of Lango and Acholi are now in progress (Morrow, R. H. and Pike, M. C., unpublished data).

Although too much importance should clearly not be given to the observation of the "blank" area, it has been thought worthwhile to carry out certain comparative studies of this area with the high incidence areas of West Nile. So far the East African Virus Research Institute have shown that the malaria rates, as measured by spleen size, are very similar, and large scale mosquito catches and viral antibody studies have failed to detect any interesting differences. A large scale prospective antibody study is just starting.

SUMMARY

The distribution in space and time of Burkitt's lymphoma patients within the West Nile District of Uganda has been studied for the period 1966–67. The space-time clustering phenomenon observed previously over the period 1961–65 in this area (Pike *et al.*, 1967) has been confirmed by this further evidence, and the hypothesis of an infective agent being involved in the aetiology of this disease is thereby strengthened.

We wish to thank Dr. M. Williams, Dr. G. Kafuko, Dr. B. Henderson and Mr. A. McCrae of the East African Virus Research Institute for help in tracing the homes of the patients. Special acknowledgement is due to both the past and the present pathologists at Makerere Medical School and in particular to Dr. D. H. Wright for carrying out the microscopy of the cases.

We wish also to thank Dr. R. H. Morrow, Dr. D. H. Wright and Dr. J. Ziegler for their help with the difficult problem of which clinical cases to include; Miss D. J. Tenwick for help with the computation; and Mr. W. Serumaga for drawing the figures.

We gratefully acknowledge the financial support of the East African Medical Research Council (to E.H.W.) and the British Empire Cancer Campaign for Research (through grants to the Kampala Cancer Registry).

### REFERENCES

Burkitt, D. P.—(1968) *W. Afr. med. J.*, **17**, 258.
Burkitt, D. P. and Wright, D. H.—(1966) *Br. med. J.*, i, 569.
Henle, G., Henle, W. and Diehl, V.—(1968) *Proc. natn. Acad. Sci., U.S.A.*, **59**, 94.
Henle, W., Diehl, V., Kohn, G., zur Hausen, H. and Henle, G.—(1967) *Science, N.Y.*, **157**, 1064.
Pike, M. C., Williams, E. H. and Wright, B.—(1967) *Br. med. J.*, ii, 395.
Williams, E. H.—(1966) *E. Afr. med. J.*, **43**, 200.

### APPENDIX A

Clinical cases:

(1) Ref.: J332. 7 years. Male. Admitted to Arua Hospital on July 7, 1966. History: weakness, loss of weight, abdominal and chest pain—no length of history given. On examination: marasmic, enlarged lymph nodes in neck, enlarged liver and spleen. On July 28 a hard swelling of right upper jaw appeared. Treated with 1200 mg. oral cyclophosphamide spread over 4 days starting July 28; and again at same dosage starting on August 5. Discharged August 10 slightly improved. Died soon after at home.

(2) Ref.: J333. 9 years. Male. Admitted to Arua Hospital on August 14, 1966. History: $1\frac{1}{2}/12$ abdominal swelling. On examination: large tumour of abdominal cavity. On August 22 a swelling of the jaw became evident. Treated with 1200 mg. oral cyclophosphamide spread over 4 days starting August 23. On August 26 a biopsy was taken of the jaw tumour but this showed only a " fragment of skin infiltrated by inflammatory cells ". There was some decrease in the size of the jaw tumour, but from September 1 onwards the child had severe convulsions without fever. On September 13 a slight exophthalmos was noted and also evidence of cranial nerve palsies. The patient left the hospital moribund on September 20 and died a few days later.

(3) Ref.: J331. 6 years. Female. Admitted to Kuluva Hospital on August 30, 1966. History: 3/7 ptosis left eye, 2/52 weakness of the legs. On examination: ptosis and slight proptosis of left eye; appeared unable to stand but no signs of spastic paraplegia; a possible small tumour of the left ovary just palpable. Treated with 1700 mg. oral cyclophosphamide spread over 6 days starting on August 31. On September 4 the child is happier, ptosis of left eye is less and she can walk. On September 29 the patient now walking and left eye open and normal, allowed to go home. On October 5 the child was visited at her home and no evidence of relapse was detected. On October 14 she returned with ptosis of the right eye. Treated with 1200 mg. oral cyclophosphamide spread over 3 days, followed by 200 mg. on October 27, 300 mg. on October 31, and 300 mg. on November 2. On November 11 there was no ptosis of either eye and she was walking normally. By November 20 the left eye ptosis had returned, with weakness of the left leg and signs of cerebral irritation. A spastic paraplegia followed and she died on November 28.

(4) Ref.: J380. 4 years. Male. Admitted to Arua Hospital on September 9, 1966. History: 1/12 swelling of right upper and lower jaw. On examination: hard tumour right upper and lower jaw. Treated with 600 mg. oral cyclophosphamide spread over 4 days starting September 14. Discharged on September 19 with tumour " fairly subsided ". Not traced.

(5) Ref.: J379. 6 years. Female. Admitted to Arua Hospital on October 28, 1966. History: 4/7 rapidly growing swelling of face. On examination: exophthalmos, ocular paralysis; tumour of right maxilla and nose. Biopsy taken on October 31 —" the tissue is largely very necrotic but would appear to have been only granulation tissue though highly vascular ". Treated with 600 mg. oral cyclophosphamide spread over 4 days starting on November 1. There was no improvement and the father took the patient home on November 5. Died about 3/52 later.

(6) Ref.: J369. 6 years. Male. Admitted to Arua Hospital on November 29, 1966. History: 2/52 swelling of lower jaw. On examination: big tumour of lower jaw, very hard on palpation. Treated with 750 mg. oral cyclophosphamide spread over 5 days starting on November 30. Swelling decreased in size, discharged December 5, 1966. Re-admitted January 14, 1967, with jaw tumour much decreased but with ptosis of right eye. Treated with 600 mg. oral cyclophosphamide spread over 4 days starting on January 16. Discharged January 20 " improved ". Admitted to Kuluva Hospital on February 24. On examination: patient unable to walk, mentally dull, with frequent nose bleeds and nasopharyngeal breathing. Treated with 1800 mg. oral cyclophosphamide. Patient also had malaria which was treated. Condition deteriorated steadily. Removed from hospital on March 5. Died few days later.

(7) Ref.: J377. 6 years. Male. Admitted to Arua Hospital on May 2, 1967. History: 2/12 swelling of right maxilla, 1/12 swelling in right submandibular region. On examination: hard swelling of right maxilla, with right upper premolar tooth loose and adjacent alveolar swelling. Treated with 1200 mg. oral cyclophosphamide spread over 4 days starting on May 8. By May 16 the swelling of the maxilla had subsided, and on this day biopsy was taken of the very large lymph gland in the submandibular region—this showed a non-lymphomatous reaction with features of tuberculosis. Treated with further 1200 mg. of oral cyclophosphamide spread over 4 days starting on May 27. Discharged on June 1. Died at home in October without returning to hospital.

(8) Ref.: K327. 10 years. Female. Admitted to Arua Hospital on May 12, 1967. History: 1/12 weakness, backache and inability to walk. On examination: swelling in right lower abdomen; enlarged right inguinal glands. Biopsy of inguinal glands—" distorted inguinal lymph node, no evidence of tumour ". Treated with 1500 mg. oral cyclophosphamide spread over 4 days starting on May 22. Abdominal swelling subsided, discharged May 29. Re-admitted June 17 with 2 large swellings in lower abdomen. Cyclophosphamide given, swellings diminished, discharged. In early July the patient developed a swelling of the right thigh and the abdominal swellings returned. Died in mid-July without returning to hospital.

(9) Ref.: J378. 3 years. Male. Admitted to Arua Hospital on June 14, 1967. History: 3/12 swelling of left part of face. On examination: very large tumour of left part of face. Treated with 500 mg. oral cyclophosphamide spread over 4 days starting on June 15. No improvement. Died June 27.

(10) Ref.: K328. 9 years. Female. Admitted to Kuluva Hospital on August 23, 1967 with swelling of both cheeks. Not thought to be Burkitt's lymphoma as the swelling subsided rapidly with antibiotic therapy. From September 3 on, the child had severe headaches and weakness on the left side of her body. A lumbar puncture done on September 22 was very bloodstained (? artefact), further lumbar puncture on September 23 showed reduced pressure, protein 300 mg. %, sugar 45–60 mg. %. By this time the patient was drowsy, had a palsy of the left arm and leg, and ankle clonus (right more than left). Two hundred milligrams intravenous and 600 mg. oral cyclophosphamide was then given with some slight effect. On October 4 a Burkitt's tumour antigen (CAN 262), prepared by the East African Virus Research Institute, gave a positive reaction to intradermal injection. Died on October 5.

(11) Patient with onset in 1965:

Ref.: J381. 5 years. Male. Admitted to Arua Hospital on January 4, 1966. History: slow growing swelling of right cheek. On examination: hard and painless large tumour right side of face involving right eye, and enlarged testes. Treated with 600 mg. oral cyclophosphamide spread over 4 days starting January 4. Discharged January 11 with tumour slightly diminished in size. Returned for further oral cyclophosphamide therapy of 750 mg. spread over 5 days on February

8 and March 16. Tumour growth controlled to some extent on both occasions. The patient was taken away on March 24. Not traced.

### APPENDIX B

The standard Knox test (Pike *et al.*, 1967) may be used to calculate the significance level of $M$ as follows: Regard all patients with date of onset in the period 1966–67 as having the same onset date (say January 1, 1967). Regard all other patients as having strictly distinct onset dates between January 1, 1961, and December 31, 1965. Then with this modified data use Knox's test with the critical time apart parameter, which defines time clustering pairs, set at 0 days, and the critical distance apart parameter set at $D$ kilometres. The significance level attained by Knox's test under these conditions is the significance level of $M$.